# Benefits of semiology taught using near-peer tutoring are sustainable

**DOI:** 10.1186/s12909-021-03086-9

**Published:** 2022-01-10

**Authors:** Benjamin Gripay, Thomas André, Marie De Laval, Brice Peneau, Alexandre Secourgeon, Nicolas Lerolle, Cédric Annweiler, Grégoire Justeau, Laurent Connan, Ludovic Martin, Loïc Bière

**Affiliations:** 1 Faculty of Health, Univ Angers, 49000 Angers, France; 2 All’Sims Centre for Simulation in Healthcare, Faculty of Health, University Hospital of Angers, 49000 Angers, France; 3 School of Medicine, University Hospital of Angers, 28 rue Roger-Amsler, 49045 Angers Cedex 01, France

**Keywords:** Near-peer tutoring, Clinical skills, Semiology, Objective structured clinical examination

## Abstract

**Background:**

Near-peer tutoring appears to be an efficient approach for teaching clinical skills. However, the clinical experience gained in the form of student medical internships may offset any interest in such tutoring programme. We then investigated the long-term benefits of this programme.

**Methods:**

This study was conducted in a medical school that experimented in near-peer tutoring for semiology intended for undergraduate medical students. Objective Structured Clinical Examinations and a written semiology test were used to assess students’ clinical skills immediately on its conclusion and repeated one and 2 years after the tutoring was completed.

**Results:**

116 students were evaluated initially (80 tutored and 36 untutored), 38 at 1 year (16 tutored and 22 untutored), 42 at 2 years (21 tutored and 21 untutored). In the global score for Objective Structured Clinical Examinations: at 1 year, the tutored group scored 14.0 ± 1.05 and the untutored group scored 11.3 ± 2.3 (*p* < 0.001), at 2 years, the tutored group scored 15.1 ± 1.5 and the untutored group scored 12.4 ± 2.2 (*p* < 0.001). We found a similar but smaller difference for the written semiology test. The difference for Objective Structured Clinical Examinations between tutored and untutored students vanished over time for cross-cutting skills.

**Conclusions:**

Near-peer tutoring in semiology for undergraduate medical students led to better results that remained with the passing of time. Though internships do allow an improvement in the clinical skills of untutored students, they did not reach the level of tutored students.

**Supplementary Information:**

The online version contains supplementary material available at 10.1186/s12909-021-03086-9.

## Background

In Medicine, adequate knowledge of semiology (science of clinical signs) is mandatory and has a significant influence on diagnosis and therapeutic decisions [1]. However, knowledge in semiology among undergraduate students appears significantly deficient, even for basic clinical skills [2, 3]. Flaws may also persist among residents and junior physicians, with an unproperly planned curriculum that fails to fill the gaps [4].

Semiology, even though it is a practical discipline, is mainly taught in the form of theoretical courses and practical workshops are rarely implemented [2]. Unfortunately, for many students, learning semiology usually begins during the clinical internship in hospitals on real patients, particularly by mimicking the clinical examination of residents and seniors. This is neither the place nor the opportune time for any first clinical experience, as unscheduled teaching may suffer from variable and questionable quality and content. Failure in senior supervision – mainly driven by lack of time [5] – constitutes another pitfall.

Near-peer tutoring (NPT) in medicine has been constantly growing, bringing with it financial advantages for the institution [6] and positive effects for student learning, especially for those that have the lowest learning skills [7]. Indeed, teaching carried out by peer tutors takes full advantage of students’ attention span, verbal and non-verbal understanding and a positive atmosphere in which to work [8]. “Cognitive congruence” [9] may indeed combine tutor proper experience as a doctor and a medical student. For the last two decades, several authors showed NPT to be as effective to faculty seniors in the quality of medical teaching [10], including knowledge, skill, and satisfaction [11].

However, the genuine effectiveness of such a tutoring program relies on maintaining the benefits over time, despite what years of internships and practicums may offer [12]. Indeed, it has been noted that, without any repetition, approximately 30% of raw knowledge was lost at 1 year and 50% at 2 years [13]. Considering the practical, personalised, and interactive nature of the NPT, clinical knowledge could be sustained over time compared to the usual academic (essentially theoretical) teaching.

Our medical school has tried out NPT for clinical examination intended for undergraduate medical students. We therefore aimed to investigate the long-term clinical benefits of NPT for semiology.

## Methods

### The study

The study was designed as a prospective interventional trial.

### Near-peer tutoring

In our institution (School of Medicine of Angers, France), academic regular lectures constituted the usual way of teaching semiology and were followed by all students.

NPT has been provided since the 2016–2017 school year. It concerned 80 s year undergraduate medical students out of promotions of about 200. NPT students were following practical sessions in small groups. This was a ten-course programme covering eight different topics (one in cardiology, pneumonology, otorhinolaryngology, life-threatening signs, uro-gynaecology and hepato-gastroenterology; two in neurology and two in rheumatology-orthopaedics). Courses were overseen by eight academics. Twenty-two tutors were recruited on a panel of 5th to 6th year volunteer medical students. All tutors previously received training in the form of courses provided by five residents involved in semiology teaching. Each course lasts two hours and includes up to four workshops; they are all supervised by a resident. Tutors conduct workshops for groups of four to five tutored.

### Objective structured clinical examination (OSCE) and written semiology test concept

OSCE is a method of assessing practical skills and is efficient at showing the benefits of NPT in semiology at the end of the programme [14]. OSCEs were overseen by academics (senior or assistant teaching staff). Each OSCE is made up of five items that are scored as either acquired (=1), partially acquired (=0.5) or non-acquired (=0). The final score is therefore between 0 and 5. OSCE topics were not revealed to students prior to the experiment.

During OSCE, the students performed physical examinations on standardised simulated patients or in manikin simulation training (for the rectal examination, Cardiopulmonary resuscitation etc.).

At the end of the NPT, each tutored student was assessed using a written theoretical exam comprising a set of fifty multiple-choice questions on semiology and a practical exam made up of five OSCEs. The scores for the five OSCEs were then assembled and presented as a 20-point score. For all the assessed students, this was their very first completion of OSCEs in their medical curriculum.

### Assessment of students in the study

Meanwhile, the academic assessment was performed at the end of a single school year, therefore across 3 different graduating classes of students. Both the written theoretical exam and OSCE were performed at the end of the tutoring program (n) and at 1 year (n + 1) and 2 years (n + 2) after it, on randomly selected panels of previously tutored and untutored students. For OSCE, 120 students from year n (out of 217), 50 from year n + 1 (out of 186) and 50 from year n + 2 (out of 228) were invited to participate in the study. For the written-test, 200 students from year n (out of 217), 50 from year n + 1 (out of 186) and 50 from year n + 2 (out of 228) were invited to participate. n + 1 and n + 2 students were randomly selected, invited and participated voluntarily.

### Statistical analysis

Total and per topic grades of tutored and untutored students were compared per year using the student t-tests. Analysis of variance (ANOVA) were run to compare scores over time. IBM SPSS V.25.0 (IBM Inc., Chicago, IL) was used for all statistical analysis. The level of statistical significance was set at 0.05.

## Results

**Fig. 1:**
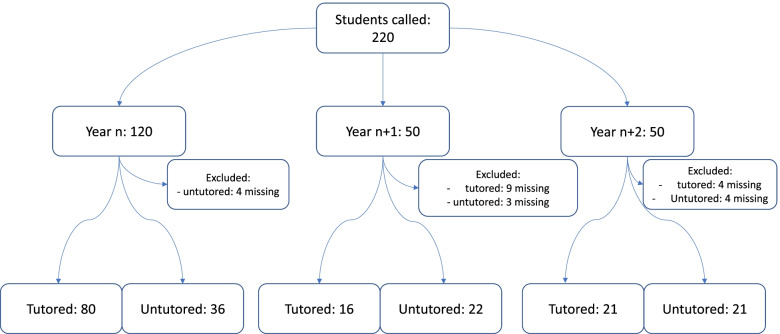
OSCE flowchart

**Fig. 2:**
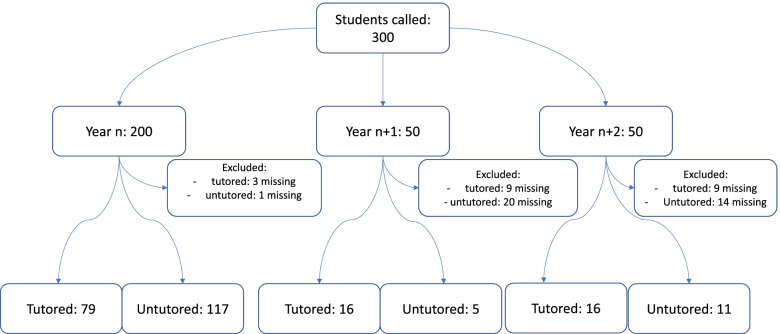
Written semiology test flowchart

**Fig. 3:**
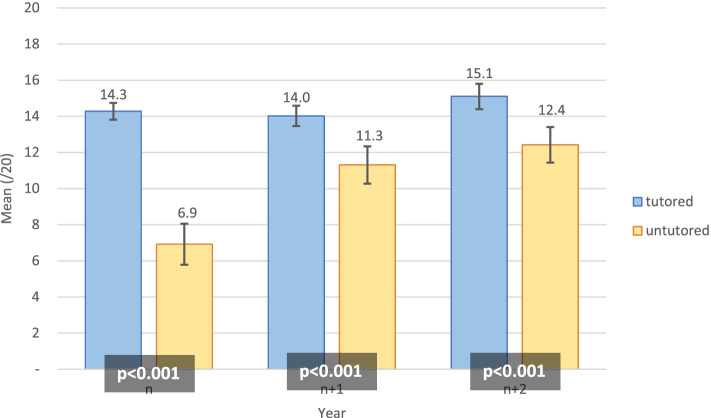
Mean of OSCE global scores (/20) among tutored and untutored students

A total of 196 students underwent OSCE and 244 students underwent the written semiology test (see Figs. 1 and 2). 24 (11%) of the invited students declined OSCE testing and 56 (19%) declined the written semiology test.

n tutored students presented higher OSCE global scores than untutored (14.3 ± 2.06 vs 6.9 ± 3.36, *p* < 0.001). n + 1 tutored students (14.0 ± 1.05 vs 11.3 ± 2.32, *p* < 0.001) and n + 2 tutored students (15.1 ± 1.54 vs 12.4 ± 2.16, *p* < 0.001) also presented higher OSCE global scores compared to untutored (Fig. 3).

Overall, the OSCE grades did not differ over time amongst tutored students (p for ANOVA = 0.14), but there was an increase amongst untutored students (p for ANOVA< 0.001). The difference between tutored and untutored students decreased over the years, from 7.4 (n) to 2.7 (n + 1) and 2.7 (n + 2) (Fig. 3).

Per-topic analysis revealed that the difference between tutored and untutored students disappeared with the passing years in neurology, otorhinolaryngology/life-threatening signs and cardiology-pneumonology. Rheumatology-orthopaedics was the only topic for which tutored were still better than untutored irrespective the year. Concerning uro-gynaecology and hepato-gastroenterology, we did not find a difference at n + 1 but we found a difference at n and n + 2 between tutored and untutored (Table 1).

**Table 1:** OSCE scores per-topic (/5) among tutored and untutored students

	n students			n + 1 students			n + 2 students		
	tutored	untutored	***p***	tutored	untutored	*p*	tutored	untutored	***p***
**Cardiology-Pneumonology**	3.81 ± 0.82	1.96 ± 1.49	< 0.001	4.03 ± 0.92	3.36 ± 1.2	0.07	4.23 ± 0.78	3.92 ± 1.0	0.27
**Neurology**	3.37 ± 1.32	1.58 ± 1.35	< 0.001	3.62 ± 0.87	2.88 ± 1.06	0.028	3.59 ± 0.75	3.04 ± 1.11	0.07
**Rheumatology-Orthopaedic**	3.31 ± 1.03	0.87 ± 1.02	< 0.001	2.5 ± 1.26	1.61 ± 1.33	0.046	2.92 ± 0.91	1.45 ± 1.26	0.001
**Otorhinolaryngology/ life-threatening signs**	2.95 ± 1.1	2.35 ± 1.34	0.013	3.46 ± 1.1	2.59 ± 1.1	0.020	3.8 ± 1.2	3.54 ± 1.1	0.46
**Uro-gynaecology -Hepato-gastroenterology**	4.38 ± 0.76	1.94 ± 1.33	< 0.001	3.9 ± 0.88	3.68 ± 1.32	0.56	4.3 ± 0.56	3.54 ± 1.16	0.010

**Fig. 4:**
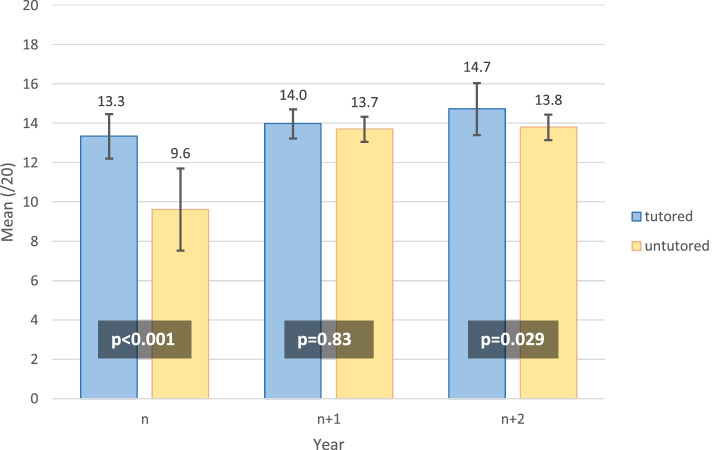
Mean of written semiology test (/20) among tutored and untutored students

Concerning the written semiology test, there was no difference at n + 1 between tutored and untutored students, but we found a difference at n and n + 2 (Fig. 4). The mean difference between tutored and untutored students varied over the years, from 3.7 (n) to 0.3 (n + 1) and 0.9 (n + 2).

## Discussion

Our study has shown that NPT provides good practical and theoretical skills in semiology. Compared to usual academic teaching, its practical counterpart appears more sustainable.

The potential reasons why NPT succeeded in improving students’ skills in semiology are numerous.

Firstly, although all students received classes in lecture halls from academics, those with NPT benefited from longer and more in-depth interactions with their peers. It is noteworthy that, in studies, OSCE results for students who received NPT did not differ from those who received personalised senior training [6, 15] or resident training [16]. In addition to greater availability, the peers are closer in age and experience than seniors and may better understand students’ levels and use more effective verbal and non-verbal communication [17]. The latter facilitates comprehension and transmission of skills and knowledge. NPT has even been shown to improve self-assessment of clinical skills and the awareness of own skills [18, 19], all under the benevolence of medical simulation.

Secondly, the purpose of semiology is practical, and NPT provides a pragmatic way to teach clinical examination [20]. It may call for visual and operative memories. The practicality of NPT has been demonstrated to provide long-term (1 year) retention of abdominal ultrasound skills [21]. We should note that our NPT programme showed better results for practical evaluation by OSCE compared to the written semiology test, even if NPT students had better written semiology test scores at baseline. Nevertheless, theoretical knowledge on semiology seemed similar among tutored and untutored students as soon as the following year (see Fig. 4), when OSCE results continued to follow a different trend (see Fig. 3). Other practical teaching, such as “The Move”, which allows the teaching of neurology through mime, showed a weak improvement in theoretical skills in semiology (at thirty months) [22]; we could expect that the better theoretical knowledge of semiology allows better practical realisation.

Thirdly, NPT may have benefited from reinforcement during a clinical internship as skills are improved when repeated, otherwise they risk deteriorating [13]. Therefore, the strength of NPT is its pragmatism, since it is put into practice almost daily during the internships. As the years went by, untutored students demonstrated gradual improvement during the course of the internship, even though this did not allow them to reach the level of the tutored students. It was related that the level of semiology is enhanced by practicum in internships [12], but that relying exclusively on them is insufficient to properly improve clinical skills [23].

Fourthly, studies noted that medical students are not highly supervised during clinical examination and interrogation [5]. In this NPT, as the students were in small groups, they were constantly observed during their practice and feedback was systematic. It was noted that the peer tutors’ feedback was well appreciated by students [24], but that one-time feedback was futile and needed to be re-iterated over time [25]. Our NPT provided regular and repeated feedback for each workshop. Feedback allows students to target the gaps that need further learning, and directly followed up their incorrect clinical examinations, in order to immediately rectify their mistakes. The benefit of increased feedback during NPT compared to that for medical doctors, has been shown to anchor clinical skills (at 4 months) [26].

Fifthly, one of the benefits of NPT is to examine healthy patients, in order to perform and understand what a normal clinical examination is. During internships, students often discover abnormal clinical examinations in disease patients without necessarily having observed the normality of the procedure. This is why some research ensures that simulated patient examination is normal, prior to assessments [14]. In addition, the pedagogical content of our NPT covered many specialities and was adapted to the students’ required knowledge, otherwise it might not have allowed long-term retention skills, as with some studies [27]. It has even been shown that tutoring can enhance an interest in the specialities taught [28], which should lead to widespread use of this practice.

Finally, the value of NPT as an early acquisition of perennial clinical skills may outreach raw competences in semiology, as it may be the catalyst to deepen knowledge of more complex skills. Indeed, our courses allow students to begin to develop their diagnostic sense, as we teach them in what context they should look for these signs. Students have increased comfort with their semiology skills when they begin internships following an early introduction of clinical skills, which may contribute to reducing their anxiety [29]. This was not explored in our study.

We observed that n + 2 students no longer presented a significant difference in OSCE scores for mainstream topics such as cardiology and pneumonology. At the time of n + 2, pathology in cardiology and pneumonology has already been taught whilst life-threatening signs and hepato-gastro-enterology are not. Moreover, it can also be pointed out that cardiology and pneumonology are mastered to a higher degree, even by untutored students, as they are pervasive during internships.

We believe that our results reflect the true level of the student clinical skills, since they were not informed in advance concerning the OSCE topics. In addition, the scores they were awarded for our assessments did not count into their academic mean, so they probably did not over-train, as they might do for higher-stakes exams, as it is sometimes reported [15].

Other studies could focus on the assessment of students’ clinical skills during their internship. While our results are encouraging, bedside teaching with direct feedback is mandatory during a medical curriculum [30], in order to anchor knowledge. It is reasonable to think that the culture of feedback in medical teaching should be encouraged.

In addition, as shown by many [31], the best way to learn a topic and retain that knowledge is to teach it. Whether there are some incremental benefits for tutors who performed evaluation as well remains to be studied. The impact of NPT on student grades should be explored in their graduate assessment.

There are some biases in this study. OCSE evaluations were not blind, as the assessors knew whether or not the students had been tutored. More, OCSE is intended to be an objective test albeit any wording may be discussed. Few students took the written exam, which probably explains the absence of a difference found in year n + 1. Moreover, untutored students who took the written semiology test were probably interested in this subject and may be among the most competent in their class.

## Conclusions

NPT in semiology for undergraduate medical students led to better results for practical and theoretical skills in semiology at the end of the training and the lessons remain with the passing of time. Though internship allowed untutored students to improve their skills, they did not achieve the level of tutored students. There was a disappearance in the difference between tutored and untutored students for topics that are practised in almost all internships.

## Supplementary Information

**Additional file 1.**

## Data Availability

The datasets used and/or analysed during the current study are available from the corresponding author on reasonable request.

## Abbreviations

NPT: Near-peer tutoring
OSCE: Objective Structured Clinical Examination
n: Second year study (year of near-peer tutoring)
n + 1: One year after near-peer tutoring
n + 2: Two years after near-peer tutoring
