# Supplementary material for: Benefits of semiology taught using near-peer tutoring are sustainable
Source: BMC Med Educ. 2022 Jan 10;22:26. doi: 10.1186/s12909-021-03086-9 (PMC8744339; doi:10.1186/s12909-021-03086-9)
Supplement: Supplementary file 1 — Additional file 1. [file 12909_2021_3086_MOESM1_ESM.docx]

### Appendices

##
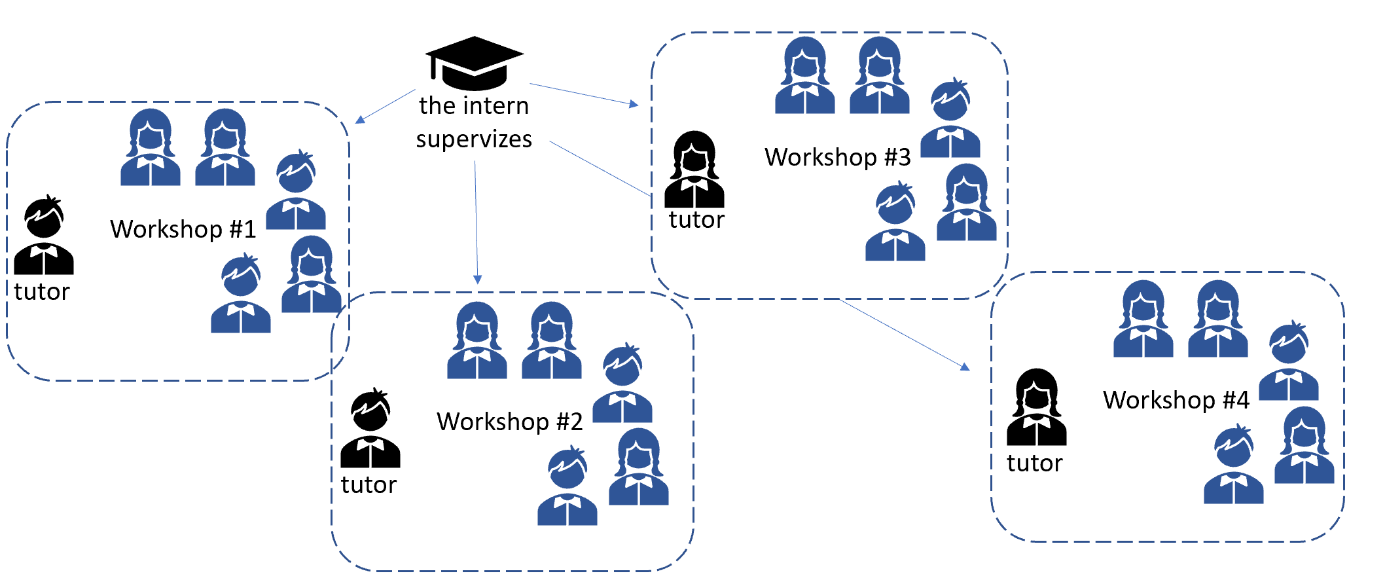
Conducting of a tutoring session

## Example session: cardiology session

| **Workshop 1 - Palpation in a healthy subject - 20 minutes**  ***Working method:***   - 4 students - 1 tutor - Students examine each other in pairs; preference for unmixed pairs - Material: 2 examination tables - Procedure: the tutor shows a typical examination with palpation. The students repeat the actions under the supervision + guidance of the tutor - Insist on the technical performance of the actions but also on the information sought and its meaning.   *Measurement of heart rate*  Radial pulse for 30 seconds  *Palpation of peripheral pulses*  The patient is in supine position.  The examiner uses the tips of the index and middle fingers.  Pulses are checked in the upper limbs (axillary, brachial, radial and ulnar) and lower limbs (femoral, popliteal, posterior tibial and pedal).   - Asymmetry or absence of a pulse indicates an arterial injury upstream.   *Search for jugular venous distention*  The patient is supine position, chest at 45°.  The head is turned to one side to better observe the jugular.   - inspection of the jugulars provides information on venous pressure.   *Search for hepato-jugular reflux*  The patient is supine position, chest at 45°.  Pressure is applied to the right hypochondrium.  Observe whether swelling of the jugulars is present   - This is a sign of right heart failure.   **Workshop 2 – Cardiac Auscultation of the healthy subject - 20 minutes**  ***Working method***:   - 4 students - 1 tutor - Students examine each other in pairs; preference for unmixed pairs - Material: 2 examination tables, 2 stethoscopes - Procedure: the tutor shows a typical examination. The students repeat the actions under the supervision + guidance of the tutor - Insist on the technical performance of the actions but also on the information sought and its meaning (normal sound; pathological sound)   *Auscultation*   - In a quiet room - Patient is shirtless - The auscultation is done in supine or seated position - Sometimes you may need to ask the patient to hold their breath to improve heart sounds listening.   *Cardiac auscultation areas*  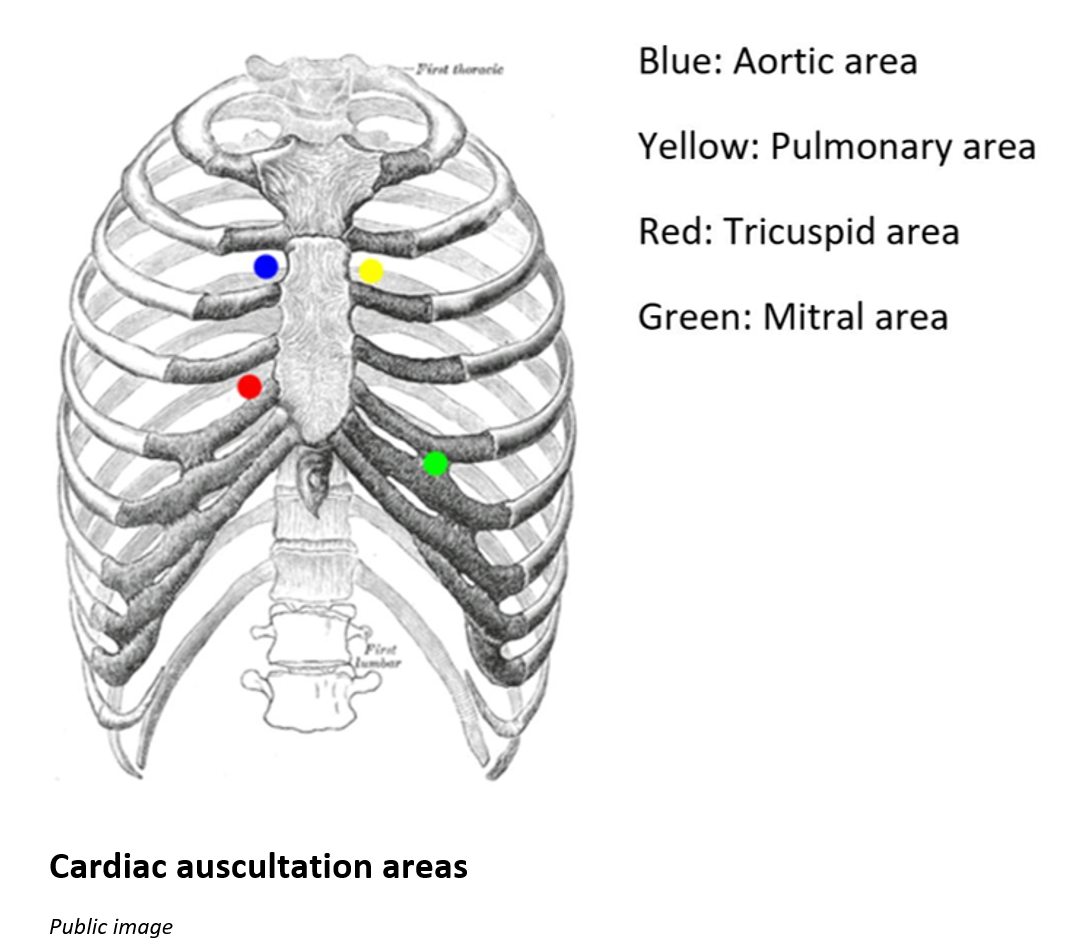  *Normal sounds*  The first sound (B1): closure of atrioventricular valves (mitral and tricuspid) at the beginning of ventricular systole  The second sound (B2): closure of aortic and pulmonary valve  **Workshop 3 - Cardiac auscultation of pathological noises - 20 minutes**  ***Working method***:   - 4 students - 1 tutor - Material: one chest auscultation dummy, one stethoscope - Procedure: the tutor recaps the main pathological noises and their meaning. Students examine the dummy to discover the main pathological sounds (mitral regurgitation, aortic stenosis, aortic regurgitation)   *Murmur of mitral regurgitation:*   - holosystolic murmur - maximum at the mitral area - axillary irradiation   *Murmur of aortic stenosis:*   - mesosystolic murmur - maximum at the 2nd right intercostal space - carotid irradiation   *Murmur of aortic regurgitation:*   - diastolic murmur - maximum at the 2nd right intercostal space - irradiation to the left edge of the sternum   **Workshop *4 - Blood pressure measurement - 20 minutes***  ***Working method***:   - 4 students - 1 tutor - Material: 2 blood pressure cuffs, 2 stethoscopes - Procedure: the tutor demonstrates the procedure. The students repeat the actions under the supervision + guidance of the tutor   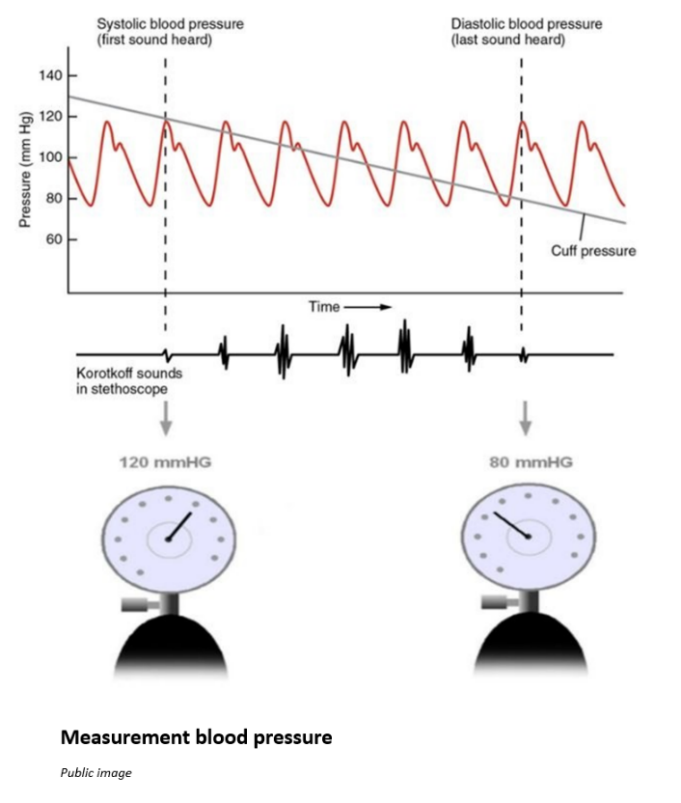  *Technique:*   1. The blood pressure cuff is placed on the biceps 2. We place the stethoscope under the cuff 3. The cuff is inflated and then deflated until you hear the sound, which corresponds to the systolic pressure 4. When the sound disappears, it corresponds to the diastolic pressure. |
| --- |

## OSCE Example

|  |
| --- |
| **Neurology topic 3:** Instruction: « Examine cranial pairs V (trigeminal: sensitivity and motricity) and VII (facial: at least 3 manoeuvres) »   \|  \| Non-acquired 0 \| Partially acquired 0.5 \| Acquired 1 \| \| --- \| --- \| --- \| --- \| \| Sensitive face testing (three territories of the V) \|  \|  \|  \| \| Chewing testing \|  \|  \|  \| \| -1 manoeuvre to test the VII* \|  \|  \|  \| \| -2 manoeuvre to test the VII* \|  \|  \|  \| \| -3 manoeuvre to test the VII* \|  \|  \|  \|   *Swelling of the cheeks OR Frowns on the eyebrows OR Raised eyebrows OR Breaking into a big smile / showing teeth OR Whistling OR Testing ear concha sensitivity OR Asking about sense of taste |
